# A white membrane beneath the inner limiting membrane of the retina in a 4-year-old child with ultrastructural evidence: a case report

**DOI:** 10.1186/s12886-018-0748-8

**Published:** 2018-03-20

**Authors:** Wenyi Tang, Ruiping Gu, Ting Zhang, Gezhi Xu

**Affiliations:** 1 Department of Ophthalmology, Eye and ENT Hospital of Fudan University, 83 Fenyang Road, Shanghai, 200031 China; 2 Shanghai Key Laboratory of Visual Impairment and Restoration, Fudan University, Shanghai, 200031 China

**Keywords:** Sub-inner limiting membrane membrane, Children, Ultrastuctural pathology, Sub-inner limiting membrane hemorrhage, Vitrectomy, Case report

## Abstract

**Background:**

Epiretinal membranes (ERMs), secondary to retinal cell proliferation on the retinal surface, usually affect patients over 50 years of age but occur rarely in children. Here we report the case of a 4-year-old patient with a unilateral sub-inner limiting membrane (sub-ILM) membrane mimicking epiretinal membrane with notable ultrastructural features indicating its possible origin from old sub-ILM hemorrhage.

**Case presentation:**

A 4-year-old boy was admitted with the complaint of poor vision in his right eye, which had been detected at school vision screening performed 6 months earlier. Fundal examination showed a feather-shaped white membrane in the macula of the right eye, and optical coherence tomography (OCT) revealed a thickened retina with a hyper-reflective band on the retinal nerve fiber layer. We suspected epiretinal membrane in the right eye, and pars plana vitrectomy with membrane peeling was performed to improve the patient’s vision. Surprisingly, the membrane was found intraoperatively to be located beneath the intact ILM; it was lifted carefully from the underlying retina as it was strongly adhered to a retinal artery of the superotemporal arcade. Postoperative scanning electron microscopy showed that the membrane consisted of hemosiderin, collagenous fibre and fibrinoid deposits. At follow-up visits, fundal examination and OCT revealed improvement in the retinal structure with disappearance of the hyper-reflective band and reduced retinal thickness. The patient’s visual acuity in the right eye was stable at 20/100 at 1 year post operation.

**Conclusions:**

The white membrane presented here was found to lie between the intact ILM and the rest of the retina, adhering firmly to the superotemporal vessel arch. Given the ultrastructural findings of the membrane and the medical history, we speculate that the sub-ILM membrane probably developed secondary to a sub-ILM hemorrhage.

## Background

Epiretinal membrane (ERM) is a nonvascular fibrocellular proliferation that occurs on the surface of the retina and causes retinal thickening and wrinkling, leading to visual impairment and metamorphopsia. ERMs are usually idiopathic and occur predominantly in patients over 50 years of age [1, 2]. In children and adolescents, however, an ERM is a very rare condition often associated with an underlying etiology such as trauma, ocular inflammation, retinal vascular disease or combined hamartoma of the retina and retinal pigment epithelium [3].

Here we report a case of a unilateral ERM-like membrane with a unique location, just beneath the inner limiting membrane (ILM), in a 4-year-old child. Scanning electron microscopy revealed hemosiderin and collagenous fibres as the main components of the membrane.

## Case presentation

**Fig. 1:**
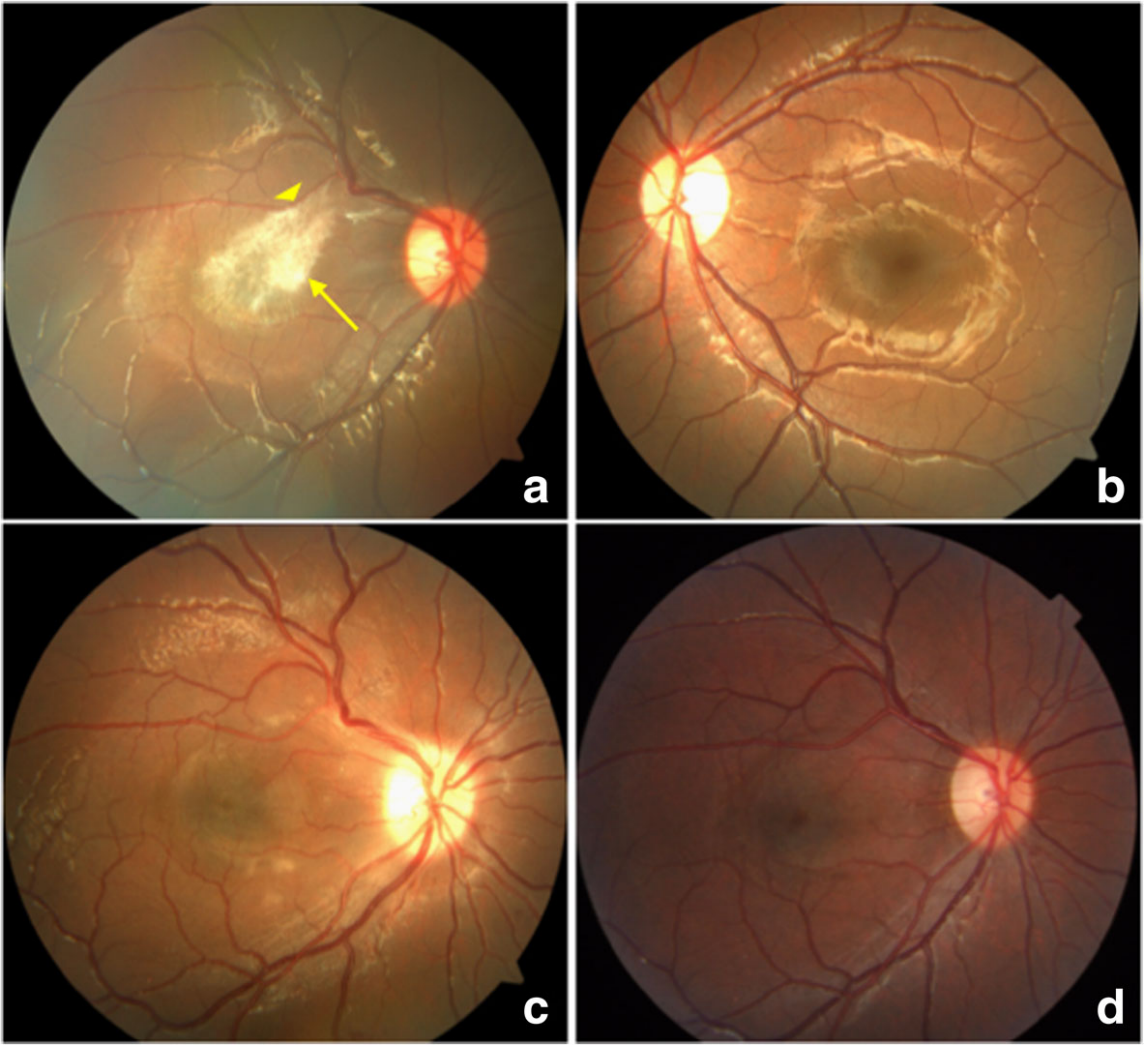
Fundus photography showing changes in the eye of a 4-year-old patient with a white membrane. **a** Preoperative fundus photography reveals a feather-shaped opaque white membrane of an approximately 1.5-papilla disc size with radial wrinkling of the retina and distorted retinal vessels in the macular region (*arrow*). The upper edge of the membrane was close to the superotemporal vessel arch (*triangle*). **b** Fundus photography of the normal left eye. **c** Fundus photography on postoperative day 1 shows the disappearance of the white membrane. **d** Fundus photography at postoperative year 1 reveals alleviation of the vessel distortion

**Fig. 2:**
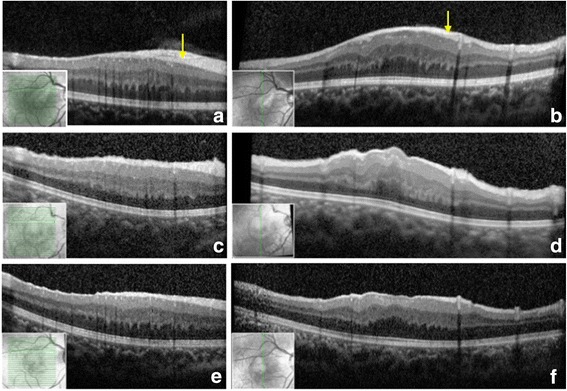
Optical coherence tomography (OCT) reveals changes in the retinal structure of the eye of a 4-year-old patient exhibiting a white membrane. **a, b** Preoperative horizontal and vertical OCT scans of the membrane show retinal thickening with a hyper-reflective band on the retinal nerve fibre layer (RNFL) (*arrow*) and disappearance of the fovea. **c, d** OCT on postoperative day 1 shows disappearance of the hyper-reflective band on the RNFL. **e, f** OCT at postoperative year 1 shows a reduction in retinal thickness

A 4-year-old boy was admitted to our centre with the complaint of poor vision in his right eye, which had been detected at school vision screening performed 6 months earlier. There was no pain, redness or any other discomfort in either eye. The patient was born at full term via uncomplicated vaginal delivery. His ocular, medication, traumatic and familial histories were unremarkable. A general physical examination was normal. An ocular examination revealed a best-corrected visual acuity (BCVA) of 20/100 in the right eye and 20/20 in the left eye. There was no evidence of strabismus. Intraocular pressure and the anterior segment of both eyes were normal. Fundal examination showed a glistening light reflex from a feather-shaped white membrane in the macular region of the right eye (Fig. 1a). The left eye was normal (Fig. 1b). The membrane of about 1.5-papilla disc size was located near the superotemporal arcade vessels, and caused radial wrinkling of the central macula and vascular distortion. The vitreous was clear, and posterior vitreous detachment (PVD) was not evident. The optic disc was unremarkable. Optical coherence tomography (OCT) revealed a hyper-reflective band on the retinal nerve fibre layer (RNFL) in the thickened retina (Fig. 2a, b). The surface of the retina was nearly smooth and uninterrupted.

**Fig. 3:**
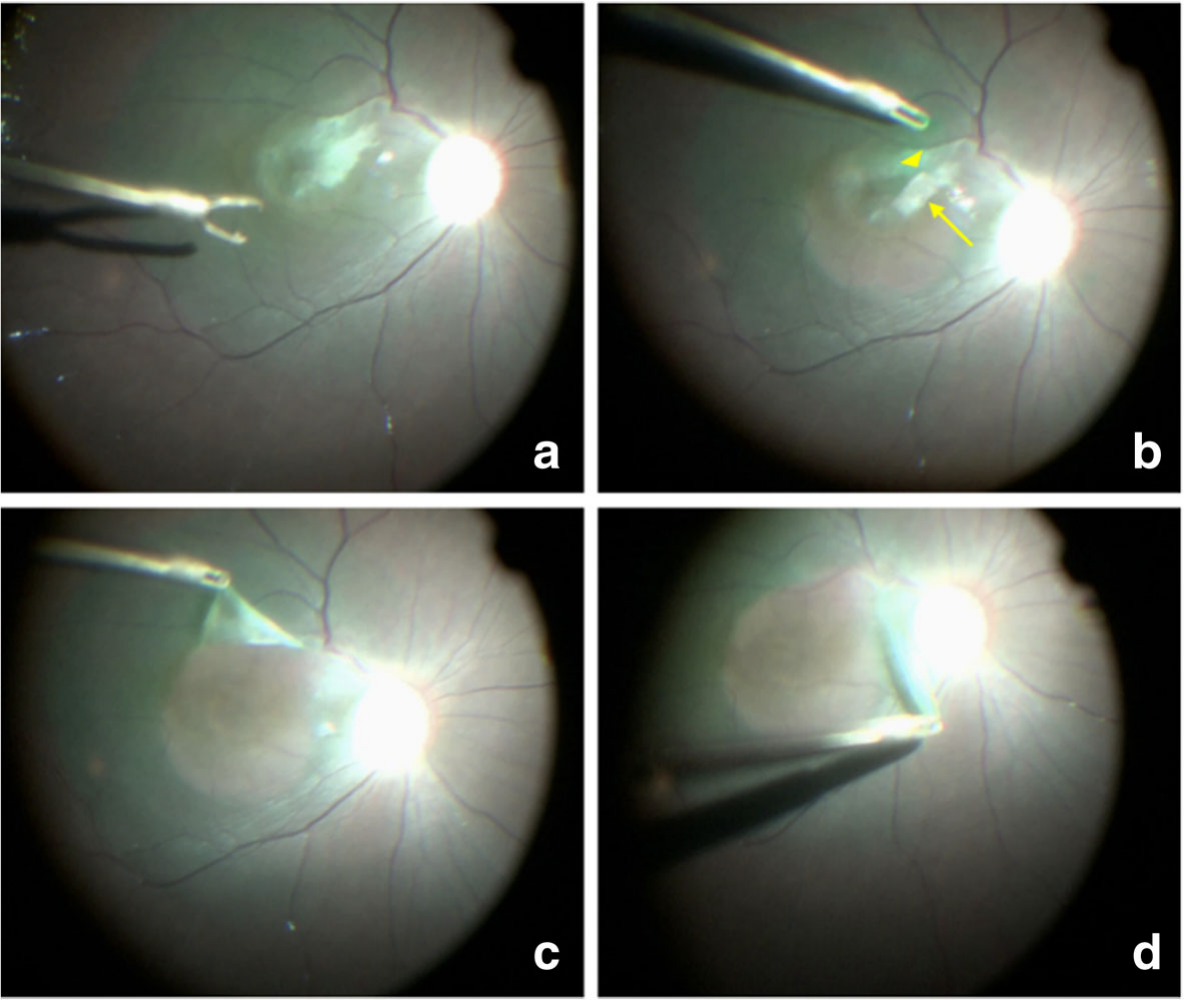
Typical video images of the surgical process. **a** Indocyanine green was used to stain the inner limiting membrane (ILM). **b** The opaque white membrane (*arrow*) was located beneath the ILM (*triangle*). **c** The thick membrane was peeled away from the rest of the retina. **d** The membrane, which was tightly adhered to the superotemporal arcade vessels, was then completely dissected from the retinal artery

**Fig. 4:**
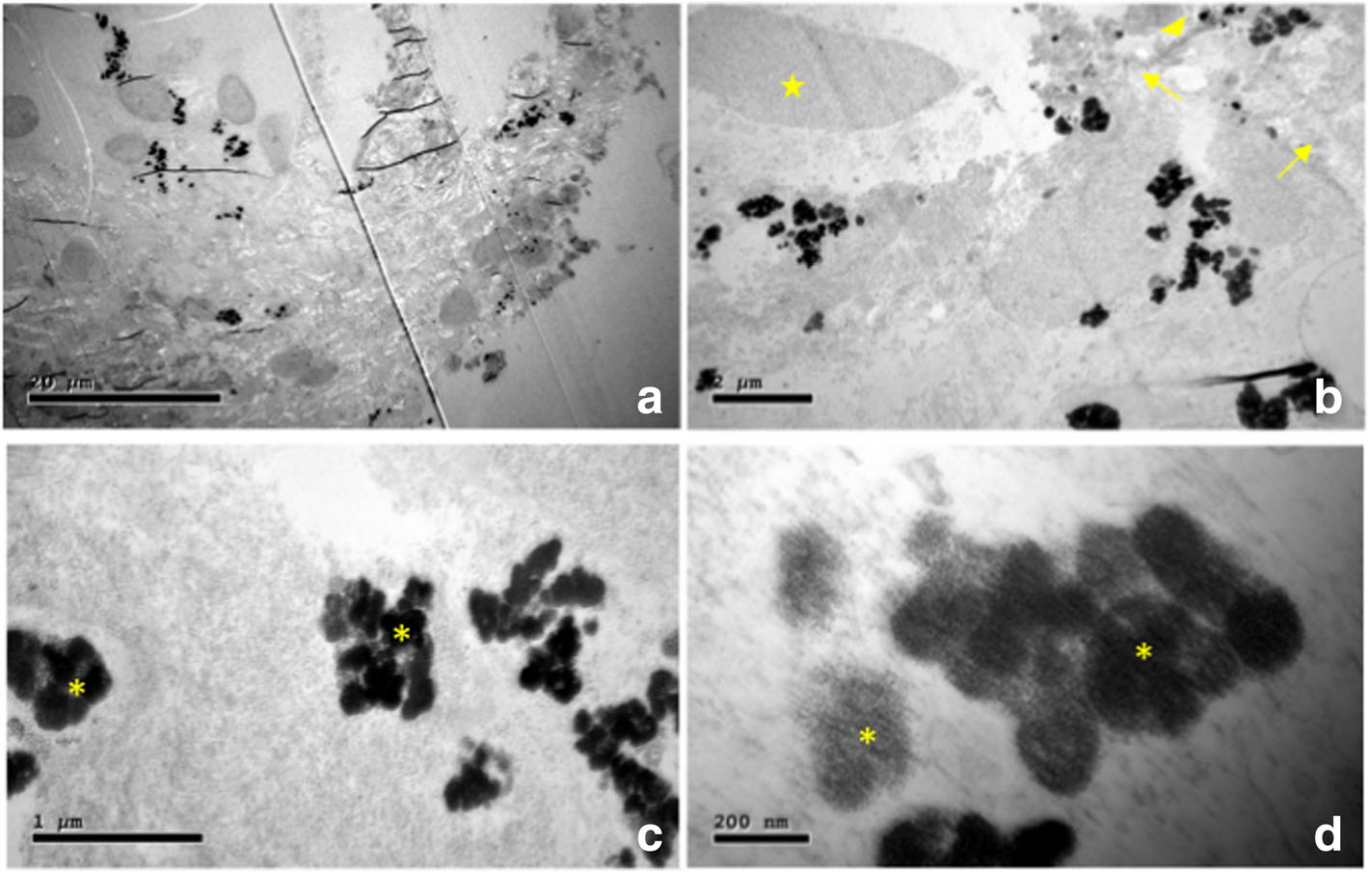
Postoperative scanning electron microscopic images of the white membrane. **a** Overview of a segment of the membrane. **b** The membrane was made up of collagenous fibres (*arrows*), fibrinoid deposits (*triangle*) and cell debris (broken nuclei, *star*). **b–d** The membrane contained abundant hemosiderin (*asterisks*)

We suspected that the decreased vision was caused by the membrane in the right eye. The patient was treated by pars plana vitrectomy using a 25 G vitrector with membrane dissection under general anaesthesia. Surprisingly, the surgeon found intraoperatively that the ILM was intact and the white membrane was located just beneath the ILM. The surgeon first peeled away the ILM following indocyanine green staining. The edge of the membrane was then lifted using forceps to separate the membrane from the underlying retina, despite its strong adhesion to a retinal artery of the superotemporal arcade (Fig. 3). There was no obvious retinal bleeding or tears. Air tamponade was used at the end of surgery. Vision training was performed 1 month after the operation. At postoperative follow-up visits, fundus photography and OCT showed successful removal of the membrane and improvement in the retinal structure with disappearance of the hyper-reflective band and reduced retinal thickness, respectively (Figs. 1c, d and 2c–f). One year later, the patient’s BCVA in the right eye was stable at 20/100. Postoperative scanning electron microscopy revealed that the membrane was composed of collagenous fibre, fibrinoid deposits and cell debris containing clusters of dense iron particles (hemosiderin) (Fig. 4). Combined with the ultrastructural results and the sub-ILM location, we speculated that the organized membrane was caused by sub-ILM haemorrhage.

## Discussion

An ERM arises secondary to the proliferation of cells along the ILM on the retinal surface. ERMs have been well characterized in adults: they are mostly idiopathic, associated with PVD and a defective ILM, and are found mainly in the elderly [1]. They are rare in adolescents and even rarer in children, as PVD is not likely to occur in these populations. The estimated incidence of ERM is 0.54 per 100,000 patients aged < 19 years [4]. The most commonly reported aetiologies are trauma (39%), uveitis (20%) and rare causes such as combined hamartoma of the retina and retinal pigment epithelium (11%); 30% of cases are idiopathic [4]. Other causes such as ocular toxocariasis, retinopathy of prematurity and Coats’ disease can also lead to secondary ERM in children [2]. However, none of these conditions were found in the case presented here.

In adult patients, ERMs generally have a cellophane-like appearance. In the present case, the fibrotic membrane was white, thick, and opaque enough to obscure the underlying retina. Preoperative OCT images depicted the membrane as a smooth hyper-reflective band located just above the RNFL, without severe involvement of the inner retinal layers, which is different to the appearance of the ERMs commonly seen in elderly patients. The widely-accepted theory of ERM formation is related to cellular proliferation and phenotypic transition on remnants of the vitreous cortex after anomalous PVD [5–7]. However, in this 4-year-old patient, the retinal surface was almost continuous and PVD was not identified. Furthermore, the membrane was located between the relatively intact ILM and the rest of the retina. As a result of this unexpected finding, we analysed the structure and composition of the membrane using scanning electron microscopy, a powerful magnification tool that revealed abundant hemosiderin deposits within the membrane. Hemosiderin is an iron-storage complex found most often in macrophages after phagocytosis of red blood cells and is especially abundant following haemorrhage [8]. Hemosiderin is hardly observed in idiopathic ERMs; previous histological studies on surgically excised ERMs have revealed the main components to be retinal glial or myoblastic retinal pigment epithelial cells [9]. Although some fibrovascular ERMs present in eyes with extensive retinal ischemia may have a primarily vascular composition, such as blood vessels with hemocytes lined by endothelial cells [10], the unremarkable retinal vasculature and medical history of this patient’s vascular disease make this case very unlikely. Taken together, it is speculated that the membrane developed after retinal sub-ILM haemorrhage and the gradual absorption and organization of the haemorrhage.

The possible causes of sub-ILM haemorrhage in children are Terson’s syndrome, shaken-baby syndrome, Valsalva maculopathy and birth-canal compression [11, 12]. However, the patient’s parents reported no history of baby-shaking, Valsalva maneuver or birth-canal compression. Nevertheless, we cannot rule out the possibility of trauma due to an accidental craniocerebral injury that could have occurred without the parents noticing. The retinal vessels of babies are not fully developed, and a surge in pressure in the intraocular veins, secondary to increased intracranial pressure during a craniocerebral injury, can cause spontaneous rupture of retinal capillaries [13]. This also partially explains the location of the membrane adjacent to the retinal vessels.

Conservative management with observation may be suitable for young patients with idiopathic ERMs, while surgical treatment may be indicated for eyes with symptomatic vision disturbances or significant anatomical changes on OCT [14, 15]. In our case, pars plana vitrectomy was performed without any complications. The BCVA, although not progressive, remained stable at 1 year postoperatively. The patient is still undergoing visual training, and the final results will be revealed at future follow-ups.

## Conclusions

To our knowledge, this case is the first report of sub-ILM haemorrhage in a child without evident retinal diseases. In contrast to the commonly seen idiopathic or secondary ERMs in terms of location and components, in this child the white membrane was composed of abundant hemosiderin deposits and located beneath the intact ILM. Therefore, we speculate that the white membrane probably developed secondary to the sub-ILM haemorrhage.

## Abbreviations

BCVA: Best-corrected visual acuity
ERM: Epiretinal membrane
ILM: Inner limiting membrane
OCT: Optical coherence tomography
PVD: Posterior vitreous detachment
RNFL: Retinal nerve fiber layer
